# Supplementary material for: Identification of Freezing-Responsive microRNAs and Their Targets in Chinese Jujube by Small RNA and Degradome Sequencing
Source: Int J Mol Sci. 2026 Sep 4;27(17):7912. doi: 10.3390/ijms27177912 (PMC13566890; doi:10.3390/ijms27177912)
Supplement: Supplementary file 1 [file ijms-27-07912-s001.zip › Supplementary Material S2/novel_miR_194.pdf]

Provisional ID	:	novel_miR_194
Score total	:	1
Score for star read(s)	:	-1.3
Score for read counts	:	0
Score for mfe	:	0.7
Score for randfold	:	1.6
Score for cons. seed	:	
Total read count	:	2332
Mature read count	:	2116
Loop read count	:	1
Star read count	:	215

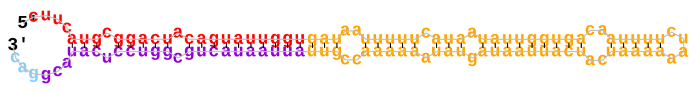

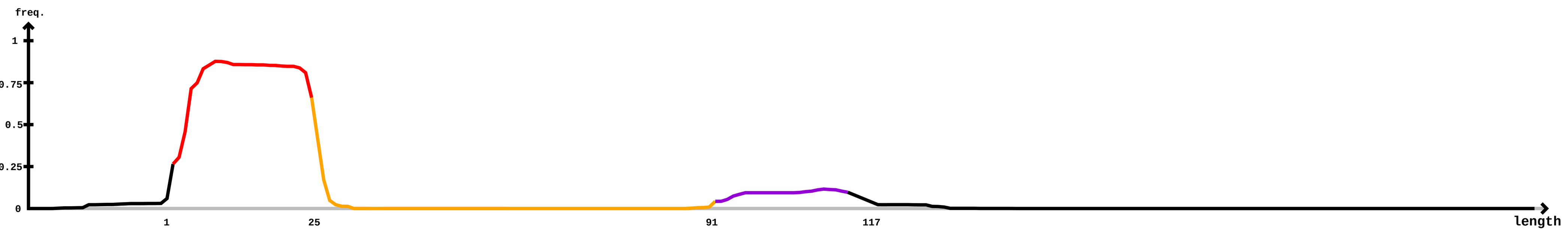

Mature Star

5'- guucauuuauggugcagacgguCUUCAUGCGGACUACAGUAUUGGUgauaauuuuucauaauauuggugacaauuuucuaaaaaaucaucauuaauaguauaaaaaaccguuauuaauacugcgguccucauacggaccauuuucaccauaaaauuucuauauauauauaaauauauauauauacauacauacaugcaaauacaauuuugaugaucuuuugguaaauuaaucaaguuagauaucacauau -3' obs

guucauuuauggugcagacggucuucaugcggacuacaguauuggugauaauuuuucauaauauuggugacaauuuucuaaaaaaucaucauuaauaguauaaaaaaccguuauuaauacugcgguccucauacggaccauuuucaccauaaaauuucuauauauauauaaauauauauauauacauacauacaugcaaauacaauuuugaugaucuuuugguaaauuaaucaaguuagauaucacauau exp

.....((((((((.(((.(((((..(((.(((((.((((((((((((..(((((.(((.(((((((((..(((((....)))))..))))))))).))).)))))..))))))))))))).))))).)))..))))).))).))))))))).....((((((((((....))))))))))........(((.((((......)))))))(((((((((......))))))..))))..........	reads	mm	sample
...cauuuauggugcagacggu......	2	0	JCK-2
...cauuuauggugcagacggu......	2	0	JCK-1
...cauuuauggugcagacggu......	1	0	JCK-3
....auuuauggugcagacggucuuca......	1	0	LT-3
....Guuuauggugcagacggucuucau......	2	1	JCK-1
....Guuuauggugcagacggucuucau......	1	1	JCK-3
......Cuauggugcagacggucuucaug......	2	1	LT-2
.......uauggugcagacggucCucaugcg......	1	1	LT-3
........auAgugcagacggucuucau......	1	1	JCK-2
........auggugcagacggucuucaug......	1	0	LT-3
........auggugcagacggucuucauAcg......	14	1	JT-1
........auAgugcagacggucuucaugcgg......	4	1	JCK-3
........auggugcagacggucuucauAcgg......	1	1	JCK-1
........auggugcagacggucCucaugcgg......	3	1	JCK-2
........auggugcagacggucuucaugcgg......	3	0	JCK-3
........auggugcagacggucuucauAcgg......	5	1	JCK-3
........auggugcagacggucuucaugcgg......	1	0	LT-3
........auAgugcagacggucuucaugcgg......	2	1	JCK-1
........auggugcagacggucuucauAcgg......	9	1	JCK-2
........auAgugcagacggucuucaugcgg......	1	1	JCK-2
..........ggugcagacggucuucaugc......	2	0	LT-3
...........gugcagacggucuucauAcgg......	2	1	JCK-2
.............gcagacgguUuucaugcggac......	2	1	JCK-2
.............gcagacgguUuucaugcggacua......	3	1	LT-1
..............cagacgguUuucaugcggacuaca......	1	1	JCK-1
..............cagacgguUuucaugcggacuaca......	3	1	JCK-3
...............agacggucuucaugcgAac......	1	1	LT-3
...............agacgguUuucaugcggacuaca......	1	1	LT-1
...............agacgguUuucaugcggacuaca......	2	1	LT-3
..................cggucuucaugcgUacuacagu......	2	1	JCK-2
....................gucAucaugcggacuacagua......	1	1	LT-3
.....................ucuucaugcggacuacaC......	1	1	JCK-2
.....................ucuucaugcggacuacaguU......	1	1	LT-3

```
                      Mature                                                                                Star
guucauuuauggugcagacggucuucaugcggacuacaguauuggugauaauuuuucauaauauuggugacaauuuucuaaaaaaucaucauuaauaguauaaaaaaccguuauuaauacugcgguccucauacggaccauuuucaccauaaaauuucuauauauauauaaauauauauauauacauacauacaugcaaauacaauuuugaugaucuuuugguaaauuaaucaaguuagauaucacauau
.....................ucuucaugcggacuacagua.................................................................................................................................................................................................... 1 0 LT-3
.....................ucuucaugcggacuacaguauu.................................................................................................................................................................................................. 6 0 JCK-3
.....................ucuucaugcggacuaUaguauu.................................................................................................................................................................................................. 1 1 JCK-2
.....................ucuucaugcggacuacaguauug................................................................................................................................................................................................. 1 0 JCK-1
.....................ucuucaugcggacuacaguauug................................................................................................................................................................................................. 1 0 JCK-2
.....................ucuucaugcggacuacaguauug................................................................................................................................................................................................. 2 0 LT-1
.....................ucuucaugcggacuaUaguauugg................................................................................................................................................................................................ 1 1 LT-3
.....................ucuucaugcggacuacaguauugU................................................................................................................................................................................................ 2 1 JCK-2
.....................ucuucaugcggacuacaguauugg................................................................................................................................................................................................ 4 0 LT-3
.....................ucuucaugcggacuaUaguauugg................................................................................................................................................................................................ 2 1 JCK-1
.....................ucuucaugcggacuaUaguauugg................................................................................................................................................................................................ 3 1 JCK-2
.....................ucuucaugcggacuacaguauugA................................................................................................................................................................................................ 3 1 JCK-2
.....................ucuucaugcggacuacaguauugg................................................................................................................................................................................................ 9 0 JCK-2
.....................uNuucaugcggacuacaguauugg................................................................................................................................................................................................ 1 1 JCK-2
.....................ucuucaugcggacuacaguauugg................................................................................................................................................................................................ 5 0 JCK-3
.....................ucuucaugcggacuacaguauugg................................................................................................................................................................................................ 5 0 JCK-1
.....................ucuucaugcggacuacaguauugg................................................................................................................................................................................................ 5 0 LCK-3
.....................ucuucaugcggacuacaguauugg................................................................................................................................................................................................ 6 0 LT-1
.....................ucuucaugcggacuaUaguauugg................................................................................................................................................................................................ 9 1 LT-1
.....................ucuucaugcggacuacaguauuggu............................................................................................................................................................................................... 5 0 JCK-2
......................cuucaugcggacuacagu...................................................................................................................................................................................................... 5 0 LT-2
......................cuucaugcggacuacagu...................................................................................................................................................................................................... 2 0 LT-3
......................cuucaugcggacuaUagua..................................................................................................................................................................................................... 1 1 LT-3
......................cuuUaugcggacuacagua..................................................................................................................................................................................................... 1 1 JCK-3
......................cuucaugcggacuacaguauC................................................................................................................................................................................................... 1 1 JCK-3
......................cuucaugcggacuacaguauC................................................................................................................................................................................................... 1 1 JCK-2
......................Auucaugcggacuacaguauug.................................................................................................................................................................................................. 1 1 LT-1
......................cuucaugcggacuacaguauug.................................................................................................................................................................................................. 13 0 LCK-3
......................cuucaugcggacuacaguauug.................................................................................................................................................................................................. 1 0 LT-2
......................cuucaugcggacuacaguauugg................................................................................................................................................................................................. 7 0 LT-2
......................cuucaugcggacuaUaguauugg................................................................................................................................................................................................. 2 1 JCK-3
......................cuucaugcggacuaUaguauugg................................................................................................................................................................................................. 7 1 JCK-2
......................cuucaugcggacuacaguauugg................................................................................................................................................................................................. 14 0 JCK-3
......................Guucaugcggacuacaguauugg................................................................................................................................................................................................. 2 1 JCK-1
......................cuucaugcggacuacaguauugg................................................................................................................................................................................................. 1 0 LT-3
......................Guucaugcggacuacaguauugg................................................................................................................................................................................................. 2 1 JCK-3
......................cuucaugcggacuacaguauugU................................................................................................................................................................................................. 2 1 JCK-1
......................cuucaugcggacuaUaguauugg................................................................................................................................................................................................. 17 1 LCK-3
......................cuucaugcggacuacaguauugU................................................................................................................................................................................................. 2 1 JCK-2
......................cuucaugcggacuacaguauuUg................................................................................................................................................................................................. 1 1 JCK-2
......................cuucaugcggacuacaguauugg................................................................................................................................................................................................. 34 0 JCK-2
......................cuucaugcggacuacaguauugg................................................................................................................................................................................................. 14 0 JCK-1
......................cuucaugcggacuaUaguauugg................................................................................................................................................................................................. 1 1 LT-1
......................cuucaugcggaAuacaguauugg................................................................................................................................................................................................. 1 1 LT-1
......................cuucaugcggacuaUaguauugg................................................................................................................................................................................................. 2 1 LT-3
......................cuucaugcggacuacaguauugA................................................................................................................................................................................................. 3 1 JCK-2
......................cuucaugcggacuaUaguauugg................................................................................................................................................................................................. 5 1 LT-2
......................cuucaugcggacuaUaguauugg................................................................................................................................................................................................. 1 1 JCK-1
......................cuucaugcggacuacaguauugg................................................................................................................................................................................................. 7 0 LT-1
......................Guucaugcggacuacaguauuggu................................................................................................................................................................................................ 8 1 JCK-2
......................cuucaugcggacuacaguauuggu................................................................................................................................................................................................ 6 0 LCK-3
......................cuucaugcggacuacaguauuUgu................................................................................................................................................................................................ 2 1 LT-3
......................cuucaugcggacuacaguauuggu................................................................................................................................................................................................ 8 0 LT-2
......................Guucaugcggacuacaguauuggu................................................................................................................................................................................................ 1 1 JCK-3
......................cuucaugcggacuacaguauuggu................................................................................................................................................................................................ 37 0 JCK-1
......................cuucauAcggacuacaguauuggu................................................................................................................................................................................................ 4 1 JCK-3
......................cuucaugcggacuacaguauuggu................................................................................................................................................................................................ 3 0 LT-1
......................cuucaugcggacuacaguauuUgu................................................................................................................................................................................................ 33 1 JCK-1
......................cuucaugcggacuacaguauuggu................................................................................................................................................................................................ 78 0 JCK-2
......................cuucauAcggacuacaguauuggu................................................................................................................................................................................................ 2 1 JCK-1
......................cuucaugcggaAuacaguauuggu................................................................................................................................................................................................ 1 1 JCK-1
......................Guucaugcggacuacaguauuggu................................................................................................................................................................................................ 2 1 JCK-1
......................cuucaugcggacuacaguauuggu................................................................................................................................................................................................ 6 0 LT-3
......................cAucaugcggacuacaguauuggu................................................................................................................................................................................................ 1 1 JCK-1
......................cuucaugcggacuacaguauuUgu................................................................................................................................................................................................ 44 1 JCK-3
......................cuucaugcggacuaAaguauuggu................................................................................................................................................................................................ 1 1 JCK-2
......................cAucaugcggacuacaguauuggu................................................................................................................................................................................................ 1 1 JCK-2
......................cuucauAcggacuacaguauuggu................................................................................................................................................................................................ 5 1 JCK-2
......................cuucaugcggacuacaguCuuggu................................................................................................................................................................................................ 1 1 JCK-1
......................cuucaugcggacuacaguauuUgu................................................................................................................................................................................................ 58 1 JCK-2
```

```
                      Mature                                                                                    Star
guucauuuauggugcagacggucuucaugcggacuacaguauuggugauaauuuuucauaauauuggugacaauuuucuaaaaaaucaucauuaauaguauaaaaaaccguuauuaauacugcgguccucauacggaccauuuucaccauaaaauuucuauauauauauaaauauauauauauacauacauacaugcaaauacaauuuugaugaucuuuugguaaauuaaucaaguuagauaucacauau
......................cuucaugcggacuacaguauuUgu............................................................................................................................................................................................................   8    1    LCK-3
......................cuucaugcggacuacaguauuggu............................................................................................................................................................................................................   61   0    JCK-3
......................cuucaugcggCcuacaguauuggu............................................................................................................................................................................................................   1    1    LT-2
.......................uucaugcggacuacaguauu...............................................................................................................................................................................................................   2    0    JCK-2
.......................uucaugcggacuacaguUuu...............................................................................................................................................................................................................   1    1    LT-2
.......................uucaugcggacuacaguauu...............................................................................................................................................................................................................   1    0    LT-1
.......................uucaugcggacuacaguauu...............................................................................................................................................................................................................   1    0    LT-3
.......................uucaugcggacuacaguauug..............................................................................................................................................................................................................   1    0    LT-1
.......................uucaugcggacuacaguauuA..............................................................................................................................................................................................................   1    1    LT-3
.......................uucaugcggacuacaguauug..............................................................................................................................................................................................................   1    0    LT-3
.......................uucaugcggacuacaguauug..............................................................................................................................................................................................................   1    0    JCK-2
.......................uucaugcggacuacaguauugg.............................................................................................................................................................................................................   1    0    LT-1
.......................uucaugcggacuacaguauugg.............................................................................................................................................................................................................   2    0    JCK-3
.......................uucaugcggacuaUaguauugg.............................................................................................................................................................................................................   1    1    LT-1
.......................uucaugcggacuaUaguauugg.............................................................................................................................................................................................................   3    1    LT-3
.......................uucaugcggacuacaguauugg.............................................................................................................................................................................................................   6    0    LT-3
.......................uucaugcggacuacaguauuggu............................................................................................................................................................................................................   8    0    JCK-3
.......................uucaugcggacuacaguauuUgu............................................................................................................................................................................................................   4    1    JCK-1
.......................uucaugcggacuacaguauuggA............................................................................................................................................................................................................   1    1    LT-1
.......................uucaugcggacuacaguauuggu............................................................................................................................................................................................................   10   0    JCK-1
.......................uucaugcggacuacaguauuggu............................................................................................................................................................................................................   2    0    LT-1
.......................uucaugcggacuacaguauuggu............................................................................................................................................................................................................   17   0    JCK-2
.......................uucaugcggacuacaguauuggu............................................................................................................................................................................................................   8    0    LT-3
.......................uucaugcggacuacaguauuUgu............................................................................................................................................................................................................   1    1    JCK-2
.......................uucaugcggacuacaguauuUgu............................................................................................................................................................................................................   3    1    JCK-3
.......................uucaugcggacuacaguauuggug...........................................................................................................................................................................................................   2    0    JCK-1
.......................uucaugcggacuacaAuauuggug...........................................................................................................................................................................................................   8    1    LCK-3
.......................uucaugcggacuacaguauuUgug...........................................................................................................................................................................................................   1    1    JCK-2
.......................uucaugcggacuacaguauuggug...........................................................................................................................................................................................................   1    0    JCK-3
.......................uucaugcggacuacaguauugguU...........................................................................................................................................................................................................   1    1    JCK-1
.......................uucaugcggacuacaguauugguU...........................................................................................................................................................................................................   1    1    JCK-3
.......................uucaugcggacuacaguauuUgug...........................................................................................................................................................................................................   1    1    JCK-3
.......................uucaugcggacuacaguauuUgug...........................................................................................................................................................................................................   3    1    JCK-1
.......................uucaugcggacuacaguauugguU...........................................................................................................................................................................................................   1    1    JCK-2
.......................uucaugcggacuacaguauuggug...........................................................................................................................................................................................................   6    0    JCK-2
........................ucaugcggacuacaguauu...............................................................................................................................................................................................................   1    0    JCK-1
........................ucaugcggacuacaguauug..............................................................................................................................................................................................................   1    0    LT-1
........................uUaugcggacuacaguauug..............................................................................................................................................................................................................   1    1    LT-3
........................ucaugcggacuacaguauug..............................................................................................................................................................................................................   4    0    JCK-3
........................ucaugcggacuacaguauug..............................................................................................................................................................................................................   4    0    JCK-2
........................ucaugcggacuacaguauug..............................................................................................................................................................................................................   4    0    JCK-1
........................ucaugcggacuacaguauug..............................................................................................................................................................................................................   11   0    LCK-3
........................ucaugcggacuacaguauugg.............................................................................................................................................................................................................   16   0    LT-3
........................ucaugcggacuacaguauugg.............................................................................................................................................................................................................   7    0    JCK-3
........................ucaugcggacuaUaguauugg.............................................................................................................................................................................................................   5    1    LT-2
........................ucaugcggacuacaAuauugg.............................................................................................................................................................................................................   3    1    JCK-1
........................ucaugcggacuacaguauuAg.............................................................................................................................................................................................................   1    1    LT-3
........................ucaugcggacuaUaguauugg.............................................................................................................................................................................................................   6    1    LT-3
........................ucaugcggacuaUaguauugg.............................................................................................................................................................................................................   6    1    JCK-2
........................ucaugcggacuaUaguauugg.............................................................................................................................................................................................................   2    1    JCK-3
........................ucaugcggacuaUaguauugg.............................................................................................................................................................................................................   4    1    LCK-3
........................ucaugcggacuacaguauugU.............................................................................................................................................................................................................   4    1    JCK-1
........................ucaugcggacuacaguauugg.............................................................................................................................................................................................................   8    0    LT-2
........................ucaugcggacuaUaguauugg.............................................................................................................................................................................................................   10   1    LT-1
........................ucaugcggacuacaguauugg.............................................................................................................................................................................................................   11   0    JCK-2
........................ucaugcggacuacaAuauugg.............................................................................................................................................................................................................   6    1    JCK-3
........................ucaugcggacuacaguauugU.............................................................................................................................................................................................................   3    1    JCK-2
........................ucaugcggacuacaguauugg.............................................................................................................................................................................................................   6    0    JCK-1
........................ucaugcggacuacaguauugU.............................................................................................................................................................................................................   8    1    JCK-3
........................ucaugcggacuacaguauugg.............................................................................................................................................................................................................   20   0    LT-1
........................ucaugcggacuacaAuauugg.............................................................................................................................................................................................................   4    1    JCK-2
........................ucaugUggacuacaguauugg.............................................................................................................................................................................................................   1    1    LT-1
........................ucaugUggacuacaguauuggu............................................................................................................................................................................................................   1    1    LT-1
........................ucaugcggacuacaguauuggu............................................................................................................................................................................................................   2    0    LT-2
........................ucaugcggacuacaguauuggA............................................................................................................................................................................................................   2    1    JCK-2
........................ucaugcggacuacaguauuggu............................................................................................................................................................................................................   6    0    LT-1
........................ucaugUggacuacaguauuggu............................................................................................................................................................................................................   2    1    LT-3
........................ucaugcggacuacaguauuAgu............................................................................................................................................................................................................   8    1    LCK-3
........................ucaugcggacuacaguauuggu............................................................................................................................................................................................................   12   0    JCK-3
........................ucaugcggacuacaguauuggu............................................................................................................................................................................................................   7    0    LCK-3
```

Mature **Star**

guucauuuauggugcagacggucuucaugcggacuacaguauuggugauaauuuuucauaauauuggugacaauuuucuaaaaaaucaucauuaaauaguauaaaaaaaccguuauuaauacugcgguccucauacggaccauuuucaccauaaaaauuucuauauauauauaaaauauauauauauacauacauacaugcaaauacaauuuugaugaucuuuugguaaauuaaucaaguuagauaucacauau

.......................ucaugcggaUuacaguauuggu...	2	1	LT-3
.......................ucauCcggacuacaguauuggu...	3	1	LT-1
.......................ucaugcggacuacaguauuggu...	7	0	JT-3
.......................ucaugcggacuacaguauuAgu...	1	1	JCK-1
.......................ucaugcggacuacaguauuAgu...	2	1	JCK-2
.......................ucaugcggacuacaguauuggu...	7	0	LT-3
.......................ucaugcggacuacaAuauuggu...	1	1	LT-3
.......................ucaugcggacuacagCauuggu...	7	1	JT-1
.......................ucaugcggacuacaguauuAgu...	1	1	LT-3
.......................ucaugcggacuaUaguauuggu...	1	1	JCK-1
.......................ucaugcggacuacaguauuAgu...	1	1	JCK-3
.......................ucaugcggacuacaguauuggu...	6	0	JT-2
.......................ucaugcggacuacaguauuggu...	5	0	JCK-1
.......................uUaugcggacuacaguauuggu...	1	1	LT-1
.......................ucaugcggacuacaguauuggu...	8	0	JCK-2
.......................uUaugcggacuacaguauuggu...	1	1	JCK-3
.......................ucaugcggacuacaAuauuggug...	1	1	LT-3
.......................ucaugcggacuacaguauuggug...	4	0	LCK-3
.......................uUaugcggacuacaguauuggug...	1	1	JCK-3
.......................ucaugcggacuacaAuauuggug...	1	1	JT-2
.......................ucaugcggacuacaguauuggug...	16	0	LT-1
.......................ucaugcggacuacaAuauuggug...	1	1	JCK-1
.......................ucaugcggacuacaguauuggug...	10	0	LT-2
.......................ucaugcggacuacaguauugguA...	1	1	LT-1
.......................ucaugcggacuacaAuauuggug...	3	1	JT-3
.......................ucaugcggacuaUaguauuggug...	3	1	LT-3
.......................ucaugcggacuaUaguauuggug...	1	1	JCK-2
.......................ucaugcggacuacaguauuggug...	5	0	LT-3
.......................ucaugcggacuaUaguauuggug...	3	1	JT-3
.......................ucaugcggaUuacaguauuggug...	2	1	LT-1
.......................ucaugcggacuaUaguauuggug...	4	1	LT-1
.......................ucaugcggacuaUaguauuggug...	1	1	JCK-3
.......................ucaugcggacuacaguauuggug...	1	0	JCK-2
.......................ucaugcggacuacaAuauuggug...	2	1	JCK-2
.......................ucaugcggacAacaguauuggug...	1	1	JCK-3
.......................ucaugcggaAuacaguauuggug...	3	1	LT-2
.......................ucaugcggacuacaAuauugguga...	2	1	JCK-1
.......................ucaugcggacuacaguauuggugU...	6	1	LCK-3
.......................ucaugcggacuaUaguauugguga...	1	1	LT-2
.......................ucaugcggacuacaguauuggugN...	1	1	LT-2
.......................ucaugcggacuacaAuauugguga...	1	1	LT-3
.......................ucaugUggacuacaguauugguga...	1	1	LT-1
.......................ucaugcggaAuacaguauugguga...	1	1	LCK-3
.......................ucaugcggacAacaguauugguga...	2	1	LT-2
.......................ucaugcggacuacaguauuggugU...	2	1	JCK-2
.......................ucaugcggacuacaguauuggugU...	1	1	LT-1
.......................ucaugcggacuacaguauugguga...	9	0	LT-3
.......................ucaugcggaUuacaguauugguga...	2	1	LT-3
.......................ucaugcggacuacaguauuggugU...	2	1	LT-3
.......................ucaugcggacuacaguauugguga...	1	0	JT-2
.......................ucaugcggacuacaguauugguga...	8	0	LT-2
.......................ucaugcggacuacaguauugguga...	8	0	LCK-3
.......................ucaugcggaUuacaguauugguga...	1	1	LT-1
.......................ucaugcggacuacaguauugguga...	2	0	JCK-2
.......................ucaugcggacuacaguauuggugU...	1	1	JCK-3
.......................ucaugcggacuacaguauugguga...	10	0	LT-1
.......................ucaugUggacuacaguauugguga...	1	1	JCK-2
.......................ucaugcggacuacaAuauugguga...	2	1	JCK-2
.......................ucaugcggacuacaguauuAguga...	3	1	LT-3
.......................ucaugcggaUuacaguauugguga...	1	1	JCK-3
........................Uaugcggacuacaguauu...	1	1	JCK-1
........................caugcggacuacaguauu...	4	0	LT-2
........................Uaugcggacuacaguauu...	1	1	JCK-3
........................caugcggacuacaguauu...	1	0	JCK-1
........................caugcggacuacaguauu...	1	0	LT-1
........................caugcggacuacaguauug...	4	0	LT-3
........................caugcggacuacaguauuA...	2	1	LT-3
........................caugcggacuacaguauug...	5	0	LT-2
........................caugcggaUuacaguauug...	2	1	LT-3
........................caugcggacuacaguauug...	6	0	LT-1

Mature   Star

guucauuuauggugcagacggucuucaugcggacuacaguauuggugauaauuuuucauaauauuggugacaauuuucuaaaaaaucaucauuaauaguauaaaaaaccguuauuaauacugcgguccucauacggaccauuuucaccauaaaauuucuauauauauauaaauauauauauauacauacauacaugcaaauacaauuuugaugaucuuuugguaaauuaaucaaguuagauaucacauau

caugcggacuaUaguauug	1	1	LT-1
caugcggacuacaguauug	1	0	JCK-2
caugcggacGacaguauug	2	1	JCK-3
caugcggacGacaguauug	1	1	JCK-2
caugcggacuaUaguauugg	4	1	LT-3
caugcggacuacaguauugU	3	1	LCK-3
caugcggacuacaguauugU	7	1	LT-2
caugcggacAacaguauugg	1	1	LT-1
caugcggacAacaguauugg	2	1	LT-2
caugcggacuacaguauugg	1	0	LT-1
caugcggacuacaguauuAg	3	1	LT-3
caugcggacuaUaguauugg	1	1	JCK-2
caugcggacuacaguauugU	1	1	LT-3
caugcggacuacaAuauugg	1	1	LT-3
Uaugcggacuacaguauugg	1	1	LT-1
caugcggacuacaguauugg	2	0	LT-3
caugcggacuaUaguauugg	8	1	LT-2
caugcggacuacaguauugg	1	0	JCK-2
caugcggacuaUaguauugg	3	1	LCK-3
caugcggacuacaguauugg	1	0	JCK-1
caugcggacuacaguauugg	1	0	JCK-3
caugcggacuacaguauugU	2	1	JCK-1
caugcggacuaUaguauugg	3	1	JCK-3
cauCcggacuacaguauuggu	1	1	JCK-1
Uaugcggacuacaguauuggu	1	1	LT-1
caugcggacuacaguauuggu	21	0	JCK-3
caugcggacuaUaguauuggu	2	1	JCK-3
caugcggaUuacaguauuggu	2	1	JCK-2
cCugcggacuacaguauuggu	1	1	LT-1
cauCcggacuacaguauuggu	4	1	JCK-2
caugcggacuacaguauuAgu	1	1	LT-1
cauCcggacuacaguauuggu	1	1	LCK-3
caugcggacuacaguauuggu	15	0	LT-1
caugcggacuacaguauuggu	5	0	JT-3
caugcggacuacaguauuggu	34	0	JCK-2
caugcggaUuacaguauuggu	1	1	LT-3
caugcggCcuacaguauuggu	1	1	JCK-2
caugcggacuacaAuauuggu	2	1	JCK-3
caugcggacuaUaguauuggu	2	1	LT-3
Uaugcggacuacaguauuggu	1	1	JCK-1
Aaugcggacuacaguauuggu	3	1	JCK-3
caugcggacuacaguauuggu	19	0	LT-3
caugcggacuaUaguauuggu	22	1	LCK-3
caugcggacuacaguauuggu	6	0	JT-2
Aaugcggacuacaguauuggu	2	1	JCK-2
cauCcggacuacaguauuggu	1	1	LT-1
caugcggacuacaAuauuggu	5	1	JCK-2
caugcggacuaUaguauuggu	5	1	LT-2
caugcggacAacaguauuggu	10	1	LCK-3
caugcggacuacaAuauuggu	4	1	LT-3
caugcggacGacaguauuggu	1	1	JCK-3
caugcggacuacaguauuggu	34	0	LCK-3
Uaugcggacuacaguauuggu	2	1	LT-3
caugcggacuacaguauuggu	33	0	LT-2
caugcggacuacaguauuggu	15	0	JCK-1
caugcggacuacaAuauuggu	5	1	LCK-3
caugcggacuacaguauuAgu	4	1	JCK-2
caugcggacuacaAuauuggu	4	1	LT-2
caugcggaUuacaguauuggu	3	1	LT-1
cauCcggacuacaguauuggu	3	1	LT-2
caugcggacuaUaguauuggu	1	1	LT-1
caugcggaUuacaguauuggu	3	1	LCK-3
caugcggacuacaguauuggug	3	0	JCK-3
caugcggacuaUaguauuggug	2	1	JCK-2
caugcggacuacaguauuggug	26	0	LCK-3
caugcggacuacaguauugguU	1	1	LT-3
caugcggacuacaguauuggug	7	0	LT-3
Uaugcggacuacaguauuggug	2	1	JCK-2
caugcggacGacaguauuggug	1	1	JCK-2
caugcggacuacaguauuggug	1	0	JCK-1

Mature   **Star**

guucauuuauggugcagacgguCUUCAUGCGGACUACAGUAUUGGUgauaauuuuucauaaauauuggugacaauuuucuaaaaaaucaucauuaauaguauaaaaaaccguuAUUAAUACUGCGGUCCUCAUACGgaccauuuucaccauaaaauuucuauauauauauaaauauauauauauacauacauacaugcaaauacaauuuugaugaucuuuugguaaauuaaucaaguuagauaucacauau			
.........................caugcggacAacaguauuggug...	1	1	LT-1
.........................caugcggacuacaguauuggug...	3	0	JCK-2
.........................Uaugcggacuacaguauuggug...	1	1	JCK-1
.........................caugcggaAuacaguauuggug...	1	1	LT-3
.........................caugcggacuaUaguauuggug...	2	1	JCK-1
.........................caugcggacAacaguauuggug...	4	1	LT-2
.........................caugcggacuacaguauuggug...	6	0	JT-2
.........................caugcggacuacaguauuggug...	13	0	JT-1
.........................caugcggacuaUaguauuggug...	2	1	LT-2
.........................caugcggacuacaguauuggug...	3	0	LT-1
.........................caugcggacuacaguauugguA...	2	1	LT-3
.........................caugcggacuaUaguauuggug...	3	1	JCK-3
.........................caugcggacuacaguauuggug...	28	0	LT-2
.........................Gaugcggacuacaguauuggug...	1	1	JCK-2
.........................caugcggacuacaguauuggug...	13	0	JT-3
.........................caugcggacuacaguauugguA...	2	1	LT-1
.........................caugcggacuaUaguauuggug...	1	1	LT-1
.........................caugcggacuacaguauugguga...	23	0	JT-1
.........................caugcggacuacaguauuAguga...	3	1	JCK-2
.........................caugcggacuacaguauugguga...	4	0	JCK-2
.........................caugcggacAacaguauugguga...	2	1	LT-2
.........................caugcggacuacaguauugguga...	3	0	JCK-3
.........................caugcggacuaUaguauugguga...	2	1	LT-3
.........................caugcggacuacaguauuAguga...	1	1	JCK-1
.........................caugcggacuaUaguauugguga...	1	1	JCK-1
.........................caugcggacuacaAuauugguga...	1	1	JCK-2
.........................caugcggacuacaguauuAguga...	3	1	LT-1
.........................caugcggacuacaguauugguga...	7	0	LT-3
.........................caugcggaUuacaguauugguga...	1	1	LT-3
.........................caugcggacuacaguauuAguga...	1	1	JCK-3
.........................caugcggaAuacaguauugguga...	1	1	JCK-2
.........................caugUggacuacaguauugguga...	4	1	LCK-3
.........................caugcggacuacaguauugguga...	4	0	LT-2
.........................caugcggacuacaguauugguga...	12	0	JT-3
.........................caugcggacuacaguauugguga...	6	0	LT-1
.........................caugcggacAacaguauugguga...	8	1	LCK-3
.........................caugcggacuacaguauugguga...	8	0	JCK-1
.........................caugcggacuacaguauugguga...	2	0	LCK-3
.........................caugcggacuacaguauugguga...	7	0	JT-2
.........................caugcggacuaUaguauugguga...	1	1	JT-3
.........................caugcggacAacaguauugguga...	3	1	LT-3
.........................caugcggacuaUaguauuggugau...	3	1	JCK-1
.........................caugcggacuaUaguauuggugau...	6	1	JCK-3
.........................caugcggacuacaguauuggugaA...	1	1	JCK-3
.........................cauAcggacuacaguauuggugau...	4	1	JCK-2
.........................caugcgAacuacaguauuggugau...	1	1	JCK-2
.........................caugcggacuacaguauuggugau...	4	0	LT-2
.........................cauAcggacuacaguauuggugau...	1	1	JCK-1
.........................caugcggacuacaguauuggugau...	2	0	JCK-1
.........................caugcggaAuacaguauuggugau...	1	1	JCK-2
.........................caugcggacuaUaguauuggugau...	3	1	LT-2
.........................caugcggacuacaguauugAugau...	1	1	JCK-2
.........................cauAcggacuacaguauuggugau...	2	1	JCK-3
.........................caugcgAacuacaguauuggugau...	1	1	JCK-3
.........................caugcggacuacaguauuggugau...	1	0	LT-3
.........................caugcggacuacaguauuggugau...	6	0	JCK-2
.........................caugcggacuaUaguauuggugau...	11	1	JCK-2
.........................caugcggacuacaguauuggugau...	3	0	JCK-3
.........................caugcggacuacaguauuggugauU...	1	1	JCK-1
..........................augcggacuacaguauugU...	1	1	LT-3
..........................augcggacuacaguauuAgu...	1	1	JCK-1
..........................augcggacuacaguauuggu...	5	0	JT-2
..........................augcggacuaAaguauuggu...	1	1	JCK-3
..........................augcggacuacaguauuggu...	15	0	JCK-3
..........................augcggacuacaguauuggu...	6	0	JCK-1
..........................augcggacuacaguauuAgu...	3	1	JCK-2
..........................augcggacuaUaguauuggu...	1	1	JCK-2
..........................augcggacuacaguauuggu...	7	0	JT-3
..........................augcggacuacaguauuggu...	4	0	LT-1
..........................augUggacuacaguauuggu...	2	1	JCK-1

```
                      Mature                                                                                  Star
guucauuuauggugcagacggucuucaugcggacuacaguauuggugauaauuuuucauaauauuggugacaauuuucuaaaaaaucaucauuaauaguauaaaaaaccguuauuaauacugcgguccucauacggaccauuuucaccauaaaauuucuauauauauauaaauauauauauauacauacauacaugcaaauacaauuuugaugaucuuuugguaaauuaaucaaguuagauaucacauau
..........................augUggacuacaguauuggu............................................................................................................................................................................................................  4    1    LCK-3
..........................augcggacuacaguauuggu............................................................................................................................................................................................................  13   0    JCK-2
..........................augUggacuacaguauuggu............................................................................................................................................................................................................  1    1    JCK-2
..........................augcggacuaUaguauuggu............................................................................................................................................................................................................  2    1    LT-1
..........................augUggacuacaguauuggu............................................................................................................................................................................................................  1    1    JCK-3
..........................augcggacuacaguauuAgu............................................................................................................................................................................................................  1    1    LT-1
..........................augcggacuacaguauuggug...........................................................................................................................................................................................................  1    0    LT-1
..........................augcggacuacaguauuggug...........................................................................................................................................................................................................  1    0    LT-3
..........................augcggacuacaguauuggug...........................................................................................................................................................................................................  1    0    JCK-2
..........................auCcggacuacaguauuggug...........................................................................................................................................................................................................  2    1    JCK-2
..........................auCcggacuacaguauuggug...........................................................................................................................................................................................................  1    1    JCK-1
..........................augcggacuacaguauuggug...........................................................................................................................................................................................................  1    0    JCK-1
..........................augcggacuacaguauuggugU..........................................................................................................................................................................................................  1    1    JCK-2
..........................augcggacuacaguauugguga..........................................................................................................................................................................................................  1    0    LT-1
..........................augcggacuacaguauuggugU..........................................................................................................................................................................................................  3    1    LT-3
..........................augcggacuacaguauuggugU..........................................................................................................................................................................................................  1    1    JCK-3
..........................augcggacuacaguauuAgugau.........................................................................................................................................................................................................  2    1    JCK-1
..........................augcggacuacaguauuggugau.........................................................................................................................................................................................................  2    0    JCK-2
..........................augcggacuacaguauuggugauG........................................................................................................................................................................................................  2    1    JCK-3
...........................ugcggacuacaguauugU.............................................................................................................................................................................................................  1    1    JCK-3
...........................ugcggacuacaguauugg.............................................................................................................................................................................................................  3    0    JCK-3
...........................ugcggacuacaguauugg.............................................................................................................................................................................................................  1    0    JCK-1
...........................ugcggaUuacaguauuggu............................................................................................................................................................................................................  1    1    JCK-1
...........................ugcggacuacaguauuggu............................................................................................................................................................................................................  10   0    JCK-2
...........................ugcggacuacaguauuggu............................................................................................................................................................................................................  4    0    JT-2
...........................ugcggaUuacaguauuggu............................................................................................................................................................................................................  1    1    LT-1
...........................ugcggacuacaAuauuggu............................................................................................................................................................................................................  10   1    LCK-3
...........................ugcggacuacaguauuAgu............................................................................................................................................................................................................  1    1    LT-2
...........................ugcggaUuacaguauuggu............................................................................................................................................................................................................  1    1    LCK-3
...........................ugcggacuacaguauuAgu............................................................................................................................................................................................................  1    1    JCK-2
...........................ugcggacuacaguauuggu............................................................................................................................................................................................................  6    0    JT-3
...........................ugcggacuacaguauuggu............................................................................................................................................................................................................  3    0    LT-3
...........................ugcggacuacaguauuggu............................................................................................................................................................................................................  3    0    JCK-1
...........................ugcggacuacaguauuggu............................................................................................................................................................................................................  10   0    LT-2
...........................ugcggacuacaguauuggu............................................................................................................................................................................................................  3    0    JCK-3
...........................ugcggacuacaguauuggug...........................................................................................................................................................................................................  9    0    LT-2
...........................ugcggacuacaguauugguA...........................................................................................................................................................................................................  1    1    LT-3
...........................ugcggacuacaguauuggug...........................................................................................................................................................................................................  3    0    JCK-3
...........................ugcggacuacaguauuggug...........................................................................................................................................................................................................  4    0    LT-3
...........................ugcggaUuacaguauuggug...........................................................................................................................................................................................................  2    1    LT-3
...........................ugcggacAacaguauuggug...........................................................................................................................................................................................................  1    1    JCK-2
...........................ugcggaUuacaguauuggug...........................................................................................................................................................................................................  1    1    LT-1
...........................ugcggaUuacaguauuggug...........................................................................................................................................................................................................  1    1    JCK-2
...........................ugcggacuacaguauuggug...........................................................................................................................................................................................................  4    0    JCK-2
...........................ugcggacAacaguauuggug...........................................................................................................................................................................................................  3    1    LT-2
...........................ugcggacuacaguauuggug...........................................................................................................................................................................................................  2    0    LT-1
...........................ugcggacuacaguauuggug...........................................................................................................................................................................................................  1    0    JCK-1
...........................ugcggacuaUaguauugguga..........................................................................................................................................................................................................  4    1    JCK-1
...........................ugcggacuacaguauugguga..........................................................................................................................................................................................................  14   0    JCK-2
...........................ugcggaUuacaguauugguga..........................................................................................................................................................................................................  1    1    LT-1
...........................ugcggacuacaguauugguga..........................................................................................................................................................................................................  13   0    JT-1
...........................ugcggacuacaguauuggugU..........................................................................................................................................................................................................  6    1    JCK-3
...........................ugcggacuacaguauugguga..........................................................................................................................................................................................................  10   0    JCK-1
...........................ugcggacuCcaguauugguga..........................................................................................................................................................................................................  1    1    JT-1
...........................ugcggaUuacaguauugguga..........................................................................................................................................................................................................  1    1    JCK-2
...........................ugcggacuacaguauuggugU..........................................................................................................................................................................................................  1    1    LCK-3
...........................ugcggacuacaguauugguga..........................................................................................................................................................................................................  1    0    LT-1
...........................ugcggacuGcaguauugguga..........................................................................................................................................................................................................  1    1    JCK-3
...........................ugUggacuacaguauugguga..........................................................................................................................................................................................................  1    1    JCK-3
...........................ugcggacuacaguauugguga..........................................................................................................................................................................................................  5    0    JCK-3
...........................ugcggacGacaguauugguga..........................................................................................................................................................................................................  1    1    JCK-2
...........................ugcggacAacaguauugguga..........................................................................................................................................................................................................  2    1    JCK-1
...........................ugcggaUuacaguauugguga..........................................................................................................................................................................................................  2    1    JCK-1
...........................ugcggacAacaguauugguga..........................................................................................................................................................................................................  1    1    LT-1
...........................ugcggacuacaAuauugguga..........................................................................................................................................................................................................  2    1    JCK-2
...........................ugcggacuaUaguauugguga..........................................................................................................................................................................................................  1    1    LT-3
...........................ugcggacuacaguauugguga..........................................................................................................................................................................................................  5    0    LT-2
...........................ugcggacGacaguauugguga..........................................................................................................................................................................................................  2    1    JCK-3
...........................ugcggaUuacaguauugguga..........................................................................................................................................................................................................  3    1    JT-3
...........................ugUggacuacaguauugguga..........................................................................................................................................................................................................  2    1    JCK-2
```

```
                      Mature                                                                                   Star
guucauuuauggugcagacgguCuucaugcggacuacaguauugguGauaaauuuuucauaauauuggugacaauuuucuaaaaaaucaucauuaauaguauaaaaaaccguuAuuaauacugcgguccucauacgGaccauuuucaccauaaaauuucuauauauauauaaauauauauauacauacauacaugcaaauacaauuuugaugaucuuuugguaaauuaaucaaguuagauaucacauau
...........................ugcggaUuacaguauugguga.........................................................................................................................................................................................................	5	1	JT-2
...........................ugAggacuacaguauugguga.........................................................................................................................................................................................................	1	1	JCK-3
...........................ugcggacuacaguauugguga.........................................................................................................................................................................................................	4	0	LT-3
...........................ugAggacuacaguauugguga.........................................................................................................................................................................................................	1	1	JCK-1
...........................ugcggaUuacaguauugguga.........................................................................................................................................................................................................	1	1	JCK-3
...........................ugcggacuacaguauuggugU.........................................................................................................................................................................................................	1	1	LT-3
...........................ugcggacuaUaguauugguga.........................................................................................................................................................................................................	1	1	LT-2
...........................ugcggacuacaAuauugguga.........................................................................................................................................................................................................	1	1	JCK-3
...........................ugcggacGacaguauugguga.........................................................................................................................................................................................................	1	1	JCK-1
...........................ugcggacAacaguauugguga.........................................................................................................................................................................................................	3	1	LT-3
...........................ugcggacuaUaguauugguga.........................................................................................................................................................................................................	1	1	LT-1
...........................ugcggacuacaguauuggugU.........................................................................................................................................................................................................	2	1	JCK-2
...........................ugcggacuaUaguauuggugau........................................................................................................................................................................................................	1	1	LT-1
...........................ugcggacuacaguauuAgugaua.......................................................................................................................................................................................................	2	1	JCK-2
...........................ugcggacuacaguauuggugauG.......................................................................................................................................................................................................	1	1	LT-2
...........................ugcggacuaUaguauuggugaua.......................................................................................................................................................................................................	3	1	JCK-2
...........................ugcggacuacaguauuggugaua.......................................................................................................................................................................................................	6	0	JT-3
...........................ugcggacuacaguauuggugaua.......................................................................................................................................................................................................	1	0	JT-2
...........................ugcggacuaUaguauuggugaua.......................................................................................................................................................................................................	2	1	JCK-1
...........................ugcggacuaUaguauuggugaua.......................................................................................................................................................................................................	2	1	JCK-3
...........................ugcggacuacaguauuggugaua.......................................................................................................................................................................................................	1	0	LT-2
...........................ugcggacuacaguauuggugauaG......................................................................................................................................................................................................	1	1	JCK-2
............................gcggacuacaguauuggu...........................................................................................................................................................................................................	6	0	JCK-2
............................gcggacuaUaguauuggu...........................................................................................................................................................................................................	2	1	JCK-1
............................gcggacuacaguauuggu...........................................................................................................................................................................................................	7	0	LCK-2
............................gcggacuaUaguauuggu...........................................................................................................................................................................................................	1	1	LT-3
............................gcggacuacaguauuggu...........................................................................................................................................................................................................	4	0	JCK-1
............................gcggacuacaguauuggu...........................................................................................................................................................................................................	1	0	LT-2
............................gcggacuacaguauuggu...........................................................................................................................................................................................................	12	0	LCK-1
............................gcggacuacaguauuggu...........................................................................................................................................................................................................	4	0	JCK-3
............................gcggacuacaguauuggu...........................................................................................................................................................................................................	1	0	LT-1
............................gcggacuaUaguauuggug..........................................................................................................................................................................................................	1	1	LT-1
............................gcggacuacaguauuggug..........................................................................................................................................................................................................	1	0	JCK-1
............................gcggaUuacaguauugguga.........................................................................................................................................................................................................	1	1	LT-1
............................gcggacuacaguauugguga.........................................................................................................................................................................................................	1	0	LT-3
............................Acggacuacaguauuggugau........................................................................................................................................................................................................	3	1	JCK-3
............................Acggacuacaguauuggugau........................................................................................................................................................................................................	1	1	JCK-2
............................Acggacuacaguauuggugau........................................................................................................................................................................................................	1	1	LT-3
............................gcggacuacaguauuggugauaGu.....................................................................................................................................................................................................	4	1	JCK-2
............................gcggacuacaguauuggugauaGu.....................................................................................................................................................................................................	1	1	LT-3
............................gcggacuacaguauuggugauaGu.....................................................................................................................................................................................................	4	1	JT-2
............................gcggacuacaguauuggugauaGu.....................................................................................................................................................................................................	2	1	JT-3
............................gcggacuacaguauuggugauaGu.....................................................................................................................................................................................................	1	1	JCK-1
.............................cggacuacaguauuggug..........................................................................................................................................................................................................	1	0	LT-1
.............................cggacuacaguauugguU..........................................................................................................................................................................................................	1	1	LT-3
.............................cggacAacaguauuggug..........................................................................................................................................................................................................	1	1	LT-1
.............................cggacuacaguauuggug..........................................................................................................................................................................................................	3	0	LT-2
.............................cggacuGcaguauuggug..........................................................................................................................................................................................................	1	1	LT-3
.............................cggacuacaguauuggugU.........................................................................................................................................................................................................	3	1	JCK-3
.............................cggacuacaguauuggugU.........................................................................................................................................................................................................	1	1	LT-2
.............................cggaUuacaguauugguga.........................................................................................................................................................................................................	4	1	JT-2
.............................cggacuacaguauuggugU.........................................................................................................................................................................................................	2	1	JCK-1
.............................cggacuacaguauugguga.........................................................................................................................................................................................................	1	0	LCK-3
.............................cggacuacaguauuggugU.........................................................................................................................................................................................................	4	1	JCK-2
.............................cggacuacaguauugguga.........................................................................................................................................................................................................	3	0	JCK-2
.............................cggaUuacaguauugguga.........................................................................................................................................................................................................	6	1	JT-3
.............................cggacuacaguauugguga.........................................................................................................................................................................................................	1	0	JCK-3
.............................cggacuacaguauugguga.........................................................................................................................................................................................................	1	0	JCK-1
.............................cggacuaUaguauuggugau........................................................................................................................................................................................................	1	1	JCK-3
.............................cggacuaUaguauuggugau........................................................................................................................................................................................................	1	1	JCK-2
.............................cggacuaUaguauuggugau........................................................................................................................................................................................................	2	1	JCK-1
.............................cggacuacaguauuggugauG.......................................................................................................................................................................................................	1	1	LT-3
.............................cggacuacaguauuggugauU.......................................................................................................................................................................................................	1	1	LT-3
.............................cggacuacaguauuggugauaGu.....................................................................................................................................................................................................	2	1	JCK-3
.............................cggacuacaguauuggugauaGu.....................................................................................................................................................................................................	2	1	JCK-2
.............................cggacuacaguauuggugauaau.....................................................................................................................................................................................................	1	0	JCK-3
.............................cggacuacaguauuggugauaau.....................................................................................................................................................................................................	3	0	JCK-2
.............................cggacuacaguauuggugauaGu.....................................................................................................................................................................................................	1	1	LCK-3
.............................cggacuacaguauugAugauaau.....................................................................................................................................................................................................	1	1	JCK-2
.............................cggacuacaguauugAugauaau.....................................................................................................................................................................................................	2	1	LT-1
```

```
                     Mature                                                                                       Star
guucauuuauggugcagacggucuucaugcggacuacaguauuggugauaauuuuucauaauauuggugacaauuuucuaaaaaaucaucauuaauaguauaaaaaaccguuauuaauacugcgguccucauacggaccauuuucaccauaaaauuucuauauauauauaaauauauauauauacauacauacaugcaaauacaauuuugaugaucuuuugguaaauuaaucaaguuagauaucacauau
.............................cggacuacaguauuggugauaGu......................................................................................................................................................................................................    4    1    JCK-1
.............................cggacuacaguauuggugauaGu......................................................................................................................................................................................................    4    1    LT-1
..................................uacaguauuggugauaGuuuu...................................................................................................................................................................................................    1    1    LT-3
.........................................................auaauauuggugaUaauuuuc............................................................................................................................................................................    1    1    LT-3
............................................................................................................cguuauuaauacugcgguccuca.......................................................................................................................    2    0    LCK-3
............................................................................................................cguuauuaauacugcgguccuca.......................................................................................................................    1    0    LT-1
............................................................................................................cguuauuaauacugcgguccuca.......................................................................................................................    1    0    JCK-2
............................................................................................................cguuauuaauacugcgguccucau......................................................................................................................    1    0    JCK-2
.............................................................................................................guuauuaauacugUgguccuca.......................................................................................................................    1    1    JCK-1
.............................................................................................................guuauuaauacugUgguccuca.......................................................................................................................    1    1    JCK-3
.............................................................................................................guuauuaauacugcgguUcucau......................................................................................................................    1    1    JCK-3
.............................................................................................................guuauuaauacugcgguUcucau......................................................................................................................    1    1    JCK-2
.............................................................................................................guuauuaauacugcgguccucauG.....................................................................................................................    3    1    JCK-3
..............................................................................................................uuauuaauacugcgguccucauGc....................................................................................................................    1    1    JCK-3
..............................................................................................................uuauuaauacugcgguccucauGc....................................................................................................................    1    1    JCK-2
...............................................................................................................uaCuaauacugcgguccucauacg...................................................................................................................    4    1    LT-2
...............................................................................................................uaCuaauacugcgguccucauacg...................................................................................................................    2    1    LT-1
...............................................................................................................uaCuaauacugcgguccucauacg...................................................................................................................    1    1    LT-3
...............................................................................................................uaCuaauacugcgguccucauacg...................................................................................................................    1    1    JCK-3
................................................................................................................auuaauacugcgguccucau......................................................................................................................    2    0    LT-3
................................................................................................................auuaauacugcgguUcucau......................................................................................................................    1    1    JCK-2
................................................................................................................auuaauacugcgguUcucau......................................................................................................................    1    1    JCK-3
................................................................................................................auuaauacugcUguccucaua.....................................................................................................................    1    1    JCK-2
................................................................................................................auuaauacugcUguccucaua.....................................................................................................................    1    1    JCK-3
................................................................................................................auuaauacugcAguccucaua.....................................................................................................................    1    1    LT-1
................................................................................................................auuaauacugcgguccucGua.....................................................................................................................    2    1    LT-3
................................................................................................................auuaauacugcgguUcucaua.....................................................................................................................    1    1    JCK-2
................................................................................................................auuaauacugcgguUcucaua.....................................................................................................................    1    1    JCK-3
................................................................................................................auuaauacugcgguUcucaua.....................................................................................................................    8    1    LCK-3
................................................................................................................aCuaauacugcgguccucauac....................................................................................................................    1    1    JCK-1
................................................................................................................aCuaauacugcgguccucauac....................................................................................................................    5    1    JCK-2
................................................................................................................auuaauacugUgguccucauac....................................................................................................................    2    1    LT-3
................................................................................................................auuaauacugcgguccucauac....................................................................................................................    1    0    LT-3
................................................................................................................auuaauacugcgguccucauaUg...................................................................................................................    1    1    JCK-2
................................................................................................................aCuaauacugcgguccucauacg...................................................................................................................    1    1    LT-3
................................................................................................................auuaauacugcgguGcucauacg...................................................................................................................    2    1    JCK-1
................................................................................................................auuaauacugcgguUcucauacg...................................................................................................................    1    1    LT-1
................................................................................................................auuaauacugcgguUcucauacg...................................................................................................................    1    1    JCK-1
................................................................................................................auuaauacugcgguGcucauacg...................................................................................................................    3    1    JCK-2
................................................................................................................aCuaauacugcgguccucauacg...................................................................................................................    5    1    LT-2
................................................................................................................auuaauacugcgguUcucauacg...................................................................................................................    6    1    JCK-3
................................................................................................................aCuaauacugcgguccucauacg...................................................................................................................    1    1    JCK-3
................................................................................................................auuaauacugcgguUcucauacg...................................................................................................................    3    1    LT-2
................................................................................................................auuaauacugcAguccucauacg...................................................................................................................    4    1    JT-1
................................................................................................................auuaauacugcgguUcucauacg...................................................................................................................    3    1    JCK-2
................................................................................................................auuaauacugcgguccucauacg...................................................................................................................    3    0    LT-3
................................................................................................................aCuaauacugcgguccucauacg...................................................................................................................    1    1    JCK-2
................................................................................................................auuaauacugcgguccucauacg...................................................................................................................    1    0    JT-1
................................................................................................................auuaauacugcAguccucauacgg..................................................................................................................    1    1    JCK-1
................................................................................................................auuaauacugcAguccucauacgg..................................................................................................................    7    1    JCK-2
................................................................................................................auuaauacugcgguccucGuacgg..................................................................................................................    2    1    LT-2
................................................................................................................auuaauacugcgguUcucauacgg..................................................................................................................    4    1    LT-2
................................................................................................................auuaauacugcAguccucauacgg..................................................................................................................    1    1    JCK-3
................................................................................................................auuaauacugcgguccucauacgA..................................................................................................................    1    1    LT-1
................................................................................................................auuaauacugcgguUcucauacgg..................................................................................................................    2    1    JCK-1
................................................................................................................auuaauacugUgguccucauacgg..................................................................................................................    2    1    LT-1
................................................................................................................auuaauacugcgguccucauacgg..................................................................................................................    1    0    JCK-2
................................................................................................................auuaauacugcgguUcucauacgg..................................................................................................................    1    1    JCK-2
.................................................................................................................uuaauacugcgguccucaua.....................................................................................................................    1    0    LT-3
..................................................................................................................uaauacugcgguccuUauacg...................................................................................................................    1    1    JCK-3
..................................................................................................................uaauacugcgguccuUauacg...................................................................................................................    2    1    JCK-2
..................................................................................................................uaauacugcgguccucauacg...................................................................................................................    1    0    LT-1
..................................................................................................................uaauacugcUguccucauacg...................................................................................................................    1    1    JCK-2
..................................................................................................................uaauacugcgguccucauacg...................................................................................................................    2    0    JCK-1
..................................................................................................................uaauacugcgguGcucauacg...................................................................................................................    1    1    LT-2
..................................................................................................................uaauacugcgguccucauacg...................................................................................................................    8    0    JCK-2
..................................................................................................................uaauacugcgguccucauacg...................................................................................................................    5    0    JCK-3
..................................................................................................................uaauacugcgguGcucauacg...................................................................................................................    3    1    JCK-3
..................................................................................................................uaauacugcgguccucauacgg..................................................................................................................    1    0    LT-1
..................................................................................................................uaauacugcgguccucauacgg..................................................................................................................    2    0    JCK-2
```

Mature Star

guucauuuauggugcagacggucuucaugcggacuacaguauuggugauaauuuuucauaauauuggugacaauuuucuaaaaaaucaucauuaauaguauaaaaaaaccguuauuaauacugcgguccucauacggaccauuuucaccauaaaauuucuauauauauauaaauauauauauauacauacauacaugcaaauacaauuuugaugaucuuuugguaaauuaaucaaguuagauaucacauau

uaauacugcgguccucauacgg	1	0	JCK-3
aauacugcgguGcucaua	1	1	LT-3
aauacugcUguccucauacg	1	1	JCK-2
aauacugcgguccucauacg	3	0	JCK-2
aauacugcgguccucauacg	1	0	JCK-3
aauacugcgguccucauacg	3	0	JCK-1
aauacugcgguAcucauacgg	2	1	JCK-3
aauacugcgguGcucauacgg	1	1	JCK-2
aauacugcgguAcucauacgg	1	1	JCK-2
aauacugcggucUucauacgg	2	1	JCK-3
aauacugcgguccucauacgg	2	0	JCK-3
aauacugcgguAcucauacgg	2	1	JCK-1
aauacugcggucUucauacgg	1	1	JCK-2
aauacugcgguccucGuacgg	2	1	LT-3
aauacugcgguGcucauacgg	1	1	JCK-3
aauacugcgguccucauacggaA	1	1	LT-3
aauacugcgguccucauacgAacc	1	1	JCK-3
aauacugcgguGcucauacggacc	5	1	JCK-2
aauacugcgguccucauacgAacc	2	1	JCK-1
aauacugcgguAcucauacggacc	2	1	JCK-3
aauacugcgguAcucauacggacc	2	1	JCK-2
aauacugcgguAcucauacggacc	2	1	LT-2
aauacugcgguGcucauacggacc	1	1	JCK-3
aauacugcgguccucauacgAacc	1	1	JCK-2
aauacugcgguGcucauacggacc	6	1	JCK-1
aauacugcgguccucauacgAacc	2	1	LT-3
aauacugcgguAcucauacggacc	1	1	JCK-1
aauacugcgguccucauacggaUca	1	1	LT-3
auacugcgguAcucauac	1	1	LT-1
auacugcgguccucauac	1	0	JCK-1
auacugAgguccucauac	3	1	LCK-3
auacugcgguccucauac	1	0	LT-3
auacugcgguccucauacg	1	0	LT-3
auacugcgguccucauacU	3	1	LT-3
auacugcgguccucauacA	2	1	LT-2
auacugcgguccucGuacgg	1	1	LT-3
auacugcgguccucauacgg	1	0	JCK-1
auacugcgguccucauacgg	1	0	LT-3
auacugcgguAcucauacgg	1	1	LT-2
auacugcgguccucauacgg	2	0	JCK-3
auacugcgguAcucauacgga	1	1	LT-3
auacugcgguAcucauacggacc	2	1	LCK-3
auacugcgguccucauacgAacc	1	1	JCK-3
auacugcgguccucauacgAacc	1	1	JCK-1
auacugcgguccucauacgAacc	1	1	LT-3
auacugcgguccucauacgAacc	2	1	LT-1
uacugcgguAcucauacgga	1	1	LT-1
uacugcgguccucauacgga	3	0	LT-3
uacugcgguccucauacAga	12	1	JT-3
uacugcgguccucauacAga	5	1	JT-2
uacugcgguGcucauacggacc	1	1	JCK-3
uacugcgguccucauacgAacc	2	1	LT-3
ccucauacggaccGuuuuc	1	1	LT-1
ccucauacggaccGuuuuca	1	1	LT-1
cUucauacggaccauuuucacc	1	1	LT-1
ccucauGcggaccauuuucacc	1	1	JCK-3
cucauacggaccGuuuuc	1	1	LT-1
cucauGcggaccauuuucacc	3	1	JCK-1
cucauGcggaccauuuucacc	3	1	JCK-2
cucauacggaccGuuuucacc	4	1	LT-3
cucauacggaccauuuuUaccau	1	1	LT-2
ucauacggaccGuuuucacc	3	1	LT-2
ucauacggaccGuuuucaccau	1	1	JCK-1
ucauacggaccauCuucaccau	1	1	LT-3
ucauacggaccGuuuucaccau	1	1	JCK-2
ucauacggaccauuuuUaccaua	2	1	JCK-2
cauacggaccGuuuucacc	2	1	JCK-3
cauacggaccGuuuucacc	1	1	JCK-2
cauacggaccGuuuucacca	2	1	LT-1
cauacggaccauuuucaccau	2	0	JCK-2

Mature   Star

```
guucauuuauggugcagacggucuucaugcggacuacaguauuggugauaauuuuucauaauauuggugacaauuuucuaaaaaaucaucauuaauaguauaaaaaaccguuauuaauacugcgguccucauacggaccauuuucaccauaaaauuucuauauauauauaaauauauauauauacauacauacaugcaaauacaauuuugaugaucuuuugguaaauuaaucaaguuagauaucacauau
.................................................................................................................................cauacggaccauuuucaccau....................................................................................................    2    0    JCK-3
.................................................................................................................................cauacggaccauCuucaccaua...................................................................................................    1    1    JCK-3
.................................................................................................................................cauacggaccauCuucaccaua...................................................................................................    1    1    JCK-1
.................................................................................................................................cauacggaccauCuucaccaua...................................................................................................    3    1    JCK-2
.................................................................................................................................cauacggaccauuuucaccaua...................................................................................................    1    0    JCK-1
.................................................................................................................................cauacggaccGuuuucaccaua...................................................................................................    1    1    LT-2
.................................................................................................................................cauacggaccauuuucaccaua...................................................................................................    1    0    LT-3
.................................................................................................................................cauacggaccauuuucaccauU...................................................................................................    2    1    LT-2
..................................................................................................................................auacggaccauuuucacc......................................................................................................    5    0    LT-2
..................................................................................................................................auacggaccauuuucaccUua...................................................................................................    1    1    LT-3
..................................................................................................................................auacggaccGuuuucaccaua...................................................................................................    3    1    JCK-2
..................................................................................................................................auacggaccGuuuucaccaua...................................................................................................    2    1    JCK-1
..................................................................................................................................auacggaccGuuuucaccaua...................................................................................................    1    1    JCK-3
....................................................................................................................................acggaccauuuucaccauaaaauu..............................................................................................    2    0    JCK-3
.............................................................................................................................................uuucaccauaaaauuucuGua........................................................................................    1    1    LT-3
```
